# Heterogeneous oxygen availability affects the titer and topology but not the fidelity of plasmid DNA produced by *Escherichia coli*

**DOI:** 10.1186/s12896-017-0378-x

**Published:** 2017-07-04

**Authors:** Karim E. Jaén, Juan-Carlos Sigala, Roberto Olivares-Hernández, Karsten Niehaus, Alvaro R. Lara

**Affiliations:** 1 Posgrado en Ciencias Naturales e Ingeniería, Universidad Autónoma Metropolita-Cuajimalpa, Av. Vasco de Quiroga 4871, Col. Santa Fe, 05348 Mexico City, Mexico; 2 Departamento de Procesos y Tecnología, Universidad Autónoma Metropolitana-Cuajimalpa, Av. Vasco de Quiroga 4871, Col. Santa Fe, 05348 Mexico City, Mexico; 3 Abteilung für Proteom- und Metabolomforschung, Fakultät für Biologie & CeBiTec, Universität Bielefeld, Universitätsstr. 25, 33615 Bielefeld, Germany

**Keywords:** Dissolved oxygen, Plasmid DNA, Microaerobic cultures, Dynamic flux balance, pDNA sequence fidelity

## Abstract

**Background:**

Dissolved oxygen tension (DOT) is hardly constant and homogenously distributed in a bioreactor, which can have a negative impact in the metabolism and product synthesis. However, the effects of DOT on plasmid DNA (pDNA) production and quality have not been thoroughly investigated. In the present study, the effects of aerobic (DOT ≥30% air sat.), microaerobic (constant DOT = 3% air sat.) and oscillatory DOT (from 0 to 100% air sat.) conditions on pDNA production, quality and host performance were characterized.

**Results:**

Microaerobic conditions had little effect on pDNA production, supercoiled fraction and sequence fidelity. By contrast, oscillatory DOT caused a 22% decrease in pDNA production compared with aerobic cultures. Although in aerobic cultures the pDNA supercoiled fraction was 98%, it decreased to 80% under heterogeneous DOT conditions. The different oxygen availabilities had no effect on the fidelity of the produced pDNA. The estimated metabolic fluxes indicated substantial differences at the level of the pentose phosphate pathway and TCA cycle under different conditions. Cyclic changes in fermentative pathway fluxes, as well as fast shifts in the fluxes through cytochromes, were also estimated. Model-based genetic modifications that can potentially improve the process performance are suggested.

**Conclusions:**

DOT heterogeneities strongly affected cell performance, pDNA production and topology. This should be considered when operating or scaling-up a bioreactor with deficient mixing. Constant microaerobic conditions affected the bacterial metabolism but not the amount or quality of pDNA. Therefore, pDNA production in microaerobic cultures may be an alternative for bioreactor operation at higher oxygen transfer rates.

**Electronic supplementary material:**

The online version of this article (doi:10.1186/s12896-017-0378-x) contains supplementary material, which is available to authorized users.

## Background

The production of plasmid DNA (pDNA) of sufficient quality for transfection to cell cultures and therapeutic purposes requires efficient and robust culture performance. Poor mass transfer can result in oxygen limitations of cultures in shake flasks [1–3], single use and large-scale bioreactors [4, 5]. In particular, dissolved oxygen tension (DOT) gradients can easily occur in large-scale bioreactors due to the limited mass transfer capacity, high oxygen demand and relatively high mixing times in such vessels [5]. Oxygen limitation strongly influences the physiology of *E. coli*, causing a metabolic rearrangement from aerobic to fermentative metabolism and leading to the accumulation of partially oxidized byproducts like organic acids and ethanol. The production of byproducts results in a decrease in the growth rate and yield of products and biomass [5]. However, less is known about the effects of oxygen on pDNA production. It has been reported that transient exposure (1.6 h) to oxygen limitation diminishes the fraction of plasmid-bearing cells in a batch culture [6]. Namdev et al. [7] reported that oxygen fluctuations created by intermittent aeration did not lead to plasmid segregation but caused a decrease in the plasmid copy number. According to another report, microaerobic conditions (DOT = 5% air sat.) did not cause plasmid loss and even increased the pDNA titer compared with aerobic conditions [8]. The effects of oxygen availability on the quality of the produced pDNA have not been properly characterized. For instance, the plasmid sequence fidelity should be verified during the production steps [9], while the pDNA topology is a quality factor required for therapeutic use [9] and transfection to cells in culture [10]. Cortassa and Aon [11] showed that plasmid supercoiling increased 6 min after shifting *E. coli* from aerobic to anaerobic conditions, and the supercoiling was maximum after 20 min of the shift. This was attributed to a decrease in the DNA relaxing activity of topoisomerase I and the constant activity of gyrase after the shift to anaerobiosis.

The above mentioned reports focused on one-step shift anaerobiosis or constant microaerobic conditions, which are not representative of large-scale cultures. In the present work, the effect of constant and oscillating DOT on the growth of *E. coli* and pDNA production was studied. Fully aerobic (DOT ≥30% air sat.) and microaerobic (DOT = 3% air sat.) conditions were used. Heterogeneous DOT conditions were simulated by the cyclic variation of the stirring speed, which resulted in DOT oscillations ranging from 100 to 0% air sat. during batch cultures. To obtain insight into the metabolic effect of the growth conditions and to better understand the cellular response, flux balance analysis was performed. The pDNA supercoiled fraction (SCF) was analyzed by agarose gels and microfluidics chips. To assess the fidelity, the produced plasmid was sequenced using Illumina Technologies.

## Methods

### Strain and plasmid

The strain used in this study was *Escherichia coli* W3110 *recA* ^−^. In such a strain, the *recA* gene was interrupted by the *cat* (chloramphenicol acetyl-transferase) gene in our laboratory according to the methodology described by Datsenko and Wanner [12]. The chloramphenicol resistance cassette was amplified from the pKD3 plasmid using the forward primer, ATGCGACCCTTGTGTATCAAACAAGACGATTAAAAATCTTCGTTAGTTTCGTGTAGGCTGGAGCTGCTTC, and the reverse primer, CAGAACATATTGACTATCCGGTATTACCCGGCATGACAGGAGTAAAAATGATGGGAATTAGCCATGGTCC. The underlined and non-underlined regions are the homologous sequences to the pKD3 plasmid and *recA* gene, respectively. The interruption of the *recA* gene was confirmed by polymerase chain reaction PCR. *E. coli* W3110 *recA* ^−^ was probed to double the SCF compared with wild-type *E. coli* W3110 (data not shown). The plasmid pVAX1 (Thermo Fisher Scientific, Waltham, MA, USA) was used as a pDNA vaccine-model in our study due to its widespread use in DNA vaccine development. It is a 3-kb high-copy number vector that confers kanamycin resistance for the selection in *E. coli*.

### Growth conditions

The medium composition (in g/L) was as follows: glucose, 10; K_2_HPO_4_, 17; KH_2_PO_4_, 5.3; (NH_4_)_2_SO_4_, 2.5; NH_4_Cl, 1.0; Citrate-Na_3_·2H_2_O, 2; MgSO_4_·7H_2_O, 1.0; Thiamine-HCl, 0.01; and trace element solution, 2 mL/L; and 50 μg/mL kanamycin sulfate. The trace element solution composition (in g/L) was ZnCl_2_, 10.5; EDTA, 5.5; CoSO_4_·7H_2_O, 1.5; MnSO_4_·H_2_O, 6.4; CuSO_4_·5H_2_O, 1.1; H_3_BO_3_, 1.5; Na_2_MoO_4_·2H_2_O, 1; FeCl_3_·6H_2_O, 51.4; and Cit-H·H_2_O, 39.9. The cultures were carried out in 500 mL of medium in a 1-L Biostat A Plus stirred-tank bioreactor (Sartorius BBI, Melsungen, Germany) at 37 °C. The pH was set at 7.2 and was controlled by the addition of 15% NH_4_OH. DOT was measured using a polarographic sensor (Hamilton, Reno, NV). The sensor was calibrated by flowing pure N_2_ (for 0% air sat.) or air (for 100% air sat.) at 1 vvm. The DOT sensor was cleaned and filled with fresh electrolyte (Oxylyte, Hamilton, Reno, NV) previous to each culture. DOT was controlled at 3 or 30% air sat. by a PI controller in the agitation cascade mode (*t*_*i*_ = 50 s; *x*_*p*_ = 140%; *t*_*D*_ = 0 s; dead band = 0.1%) using MFCS/DA software (Sartorius BBI, Melsungen, Germany). For oscillated DOT cultures, the stirrer speed was shifted from 100 to 1200 rpm every 10 min. Air was supplied at 1 vvm for aerobic and oscillated cultures and at 0.25 vvm for microaerobic cultures. Off-gas composition was monitored on-line through a BlueInOne Ferm (BlueSens, Herten, Germany) gas analyzer. Three independent cultures under each condition were performed.

### Off-line analyses

The biomass concentration was determined as the dry cell weight. Extracellular metabolites were quantified from filtered (0.2 μm cellulose acetate membranes) supernatants. Glucose and ethanol were quantified in a YSI 2700 biochemistry analyzer (YSI Inc., OH, USA). Organic acids were analyzed by HPLC using a Bio-Rad Aminex HPX-87H column (Bio-Rad Laboratories Inc., CA, USA) at 50 °C and 0.4 mL/min of 5 mM H_2_SO_4_, and a UV detector set at 210 nm.

### OTR, CTR and RQ calculations

From the off-gas composition data, OTR and CTR were calculated as follows:

1 $$ OTR=\frac{P_i}{R{ T}_i}\frac{F_i}{V_L}\left({y}_{O_2, i}-{R}_I{y}_{O_2, o}\right) $$

2 $$ C T R=\frac{P_i}{R{ T}_i}\frac{F_i}{V_L}\left({R}_I{y}_{C{ O}_2, o}-{y}_{C{ O}_2, i}\right) $$

3 $$ {R}_I=\frac{1 - {y}_{O_2, i}-{y}_{CO_2, i} - {y}_{H_2 O, i}}{1-{y}_{O_2, o} - {y}_{CO_2, o}-{y}_{H_2 O, o}} $$

where:

- OTR, oxygen transfer rate (mmol/L/h);
- CER, carbon dioxide volumetric production rate (mmol/L/h);
- *p*, absolute pressure of the gaseous stream, in bar;
- *F*, volumetric flow rate of the gaseous stream (L/h);
- R, ideal gas constant (0.0821 bar L K^−1^ mol^−1^);
- *V*_*L*_, liquid volume (L);
- *T*, temperature of the gaseous stream (K);
- ${y}_{O_2}$, oxygen molar fraction in the gaseous stream;
- ${y}_{CO_2}$, carbon dioxide molar fraction in the gaseous stream;
- ${y}_{H_2 O}$, water molar fraction in the gaseous stream;
- *R*_*I*_, inert ratio;
- *i* and *o* subscripts denote at inlet and outlet, respectively.

The specific rates *q*_*O2*_ and *q*_*CO2*_ were determined by plotting OTR and CER, respectively, against the biomass concentration (X), and obtaining the slope from the line of best fit by the least squares method. Only data from the period where DOT was constant was used.

For the oscillated cultures, where such conditions were not met, the global specific uptake rates were calculated as follows:

4 $$ {q}_{O_2}=\frac{\int OTR\ (t) dt}{X} $$

5 $$ {q}_{O_2}=\frac{\int CTR(t) dt}{X} $$

For all cases, RQ was calculated as the ratio,

6 $$ R Q=\frac{\int CTR(t) dt}{\int OTR(t) dt} $$

Numerical integrations were computed by SigmaPlot 12.5 Area Below Curves Macro.

### Metabolic fluxes estimation

#### Flux balance analysis (FBA)

The flux distribution in the metabolic network was calculated based on a linear programming algorithm, using the specific uptake rates and specific production rates as inputs for the calculations (Flux Balance Analysis) [13]. The network studied here comprised 103 reactions and 76 metabolites (intracellular and extracellular) covering glycolysis, pentose phosphate, tricarboxylic acid (TCA) cycle, and mixed-acid fermentation pathways. It included reactions for transport and exchange, plus biomass and plasmid synthesis objective functions. The extracellular exchange of pyruvate and malate were considered to be inactive. Simulations were run using MatLab. Fluxes were constrained according to the reversibility of the reactions. The reaction rate of formate hydrogen-lyase (FHL) was constrained to 0 in all simulations. Formate decomposition into CO_2_ and H_2_ seems not to be a plausible reaction because the mineral media was devoid of selenium and nickel, which are required for the catalytic activity of FHL [14].

#### Sensitivity analysis

Sensitivity analysis was performed to evaluate the biomass function response to changes in the two measured input fluxes (${q}_{O_2}$and *q*_*s*_). Simulations to maximize the biomass by changing ${q}_{O_2}$ and *q*_*S*_ fluxes were run. The results are provided as Additional files.

#### Time-point flux balance analysis

To analyze the metabolic effect of the non-steady state condition of the oscillated cultures, flux balance analysis was carried out for biomass maximization at every time-point of the culture. The O_2_ and CO_2_ fractions in the exhaust gas were used as data inputs. O_2_ and CO_2_ fluxes were calculated by dividing CTR and OTR by the biomass concentration. The latter was obtained from a second-order polynomial fit to the experimental biomass cell dry weight concentration and time data. Spreadsheets detailing the calculations, constraints and results, as well as the Matlab code, are provided as Additional files.

#### pDNA analysis

pDNA was isolated and purified from 5.8 mg of wet biomass using the QIAprep Spin Miniprep Kit (Qiagen, Hilden, Germany), and recovered in 70 μL of EB buffer at 70 °C. Such procedure enabled to maximize the amount of pDNA extracted from cells (data not shown), whereas SCF is not expected to be influenced, as indicated by the manufacturer. The extracted pDNA was quantified in a Nanodrop 2000 system. The SCF was determined from the image analysis of 0.8% agarose gels pre-stained with SYBR green safe (Invitrogen, Carlsbad, CA, USA). Additionally, pDNA samples were analyzed by chip-electrophoresis in a Bioanalyzer 2100 system (Agilent Technologies, Santa Clara, CA, USA) using the Agilent DNA 7500 kit. Prior to the run, the samples were concentrated by lyophilization (500–900 ng/μL) to obtain fluorescence signals within the reading capabilities of the equipment. pDNA samples from the master cell bank and the different production conditions were sequenced. pDNA sequencing and assembly were performed using the Genome Analyzer II (Illumina, CA, USA) and Velvet 1.2.0 program, respectively. The resulting assembled sequences were aligned with the reported reference sequence of the pVAX1 plasmid (Thermo Fisher Scientific, Waltham, MA, USA) using the BLASTN 2.6.1+ suite [15] (NCBI, USA). Additionally, sequences were also aligned by ClustalW and LALING [16] to compare and corroborate the results obtained from the different algorithms. pDNA sequencing and assembly were performed at the Unidad de Secuenciación Masiva of the Universidad Nacional Autónoma de México and the Laboratorio de Servicios Genómicos of the Laboratorio Nacional de Genómica para la Biodiversidad, México.

## Results

### DOT control profiles and on-line measurements

**Fig. 1:**
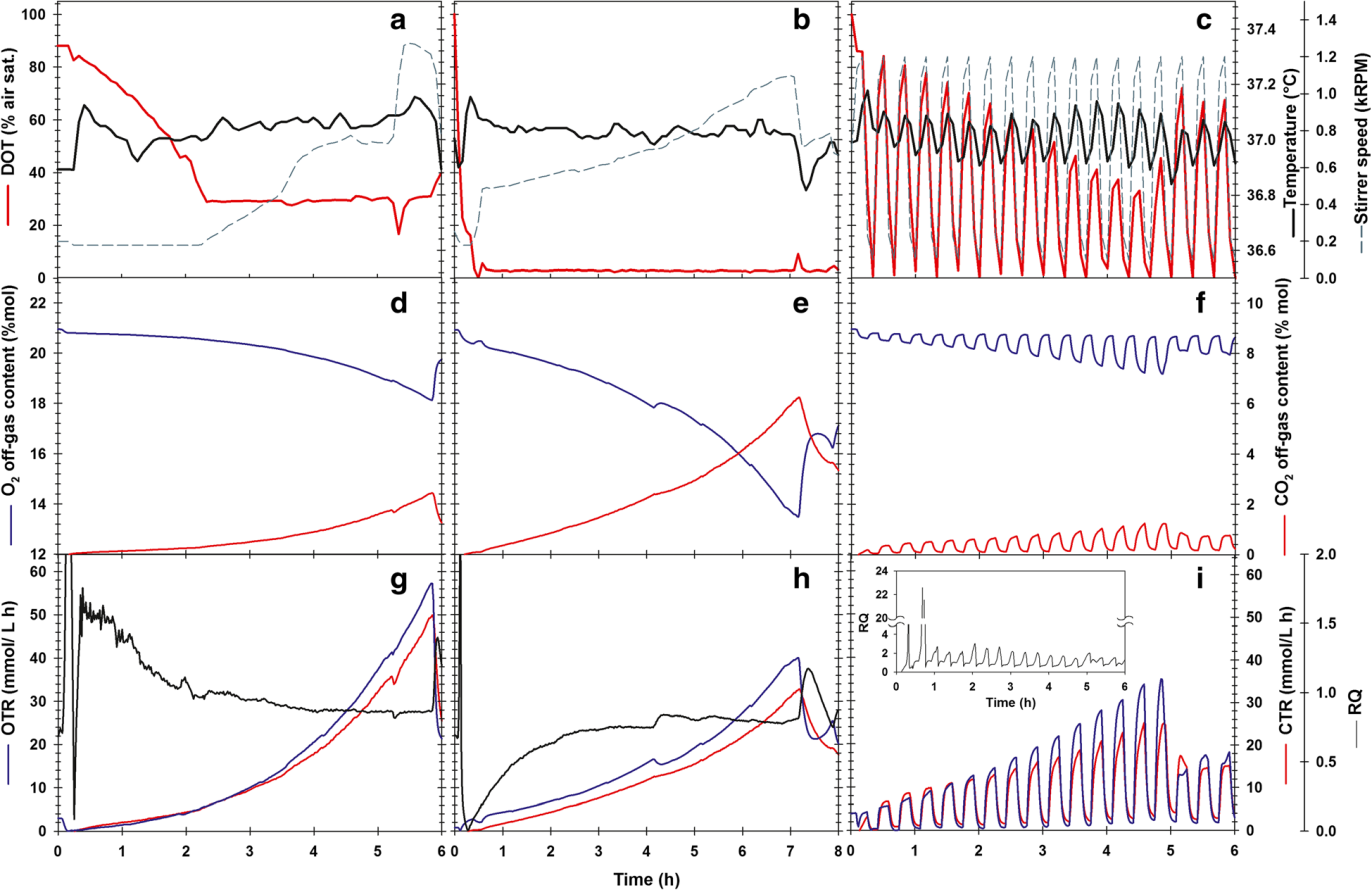
On-line monitoring data of the cultures. **a**-**c** DOT, temperature and stirrer speed: **d**-**f** exhaust-gases composition; **g**-**i** and OTR, CTR and RQ. *Left panel*: aerobic cultures; *middle panel*: microaerobic cultures; *right panel*: oscillated DOT cultures

The DOT profiles, O_2_/CO_2_ off-gas content and temperature measurements of the cultures are shown in Fig. 1. In aerobic cultures, the DOT was higher than 30% air sat. during the first 2 h and was controlled to 30% thereafter (Fig. 1a). The DOT was effectively controlled at 3% air sat. in microaerobic cultures (Fig. 1b). In oscillated cultures, the stirrer speed shifts from 100 to 1200 RPM every 10 min caused DOT oscillations with decreasing amplitude from 0 to 90% from the beginning to 0–40% by the end of the culture (Fig. 1c). Interestingly, irregular temperature variations were seen in aerobic and microaerobic cultures, while cyclic oscillations were observed under oscillatory DOT (Fig. 1a-c). Such temperature oscillations followed the pattern of the DOT oscillations. The off-gas from the microaerobic cultures was more exhausted in O_2_ and enriched in CO_2_ than in the aerobic cultivation, whereas the opposite was observed under oscillatory DOT (Fig. 1d-f). Furthermore, the O_2_ and CO_2_ content showed an oscillatory pattern in synchrony with DOT oscillations (Fig. 1f). The highest OTR and CTR occurred in aerobic cultures; however, in microaerobic cultures, they were reduced in 40–50% (Fig. 1g-i). In aerobic cultures, the RQ was 0.93 ± 0.03; however, in microaerobic cultures, RQ dropped to 0.76 ± 0.06, indicating that less carbon was fully oxidized to CO_2_. Under oscillating DOT, RQ was greater than 1, pointing to a prevalent fermentative metabolism.

### Kinetic profile under constant and oscillating DOT conditions

**Fig. 2:**
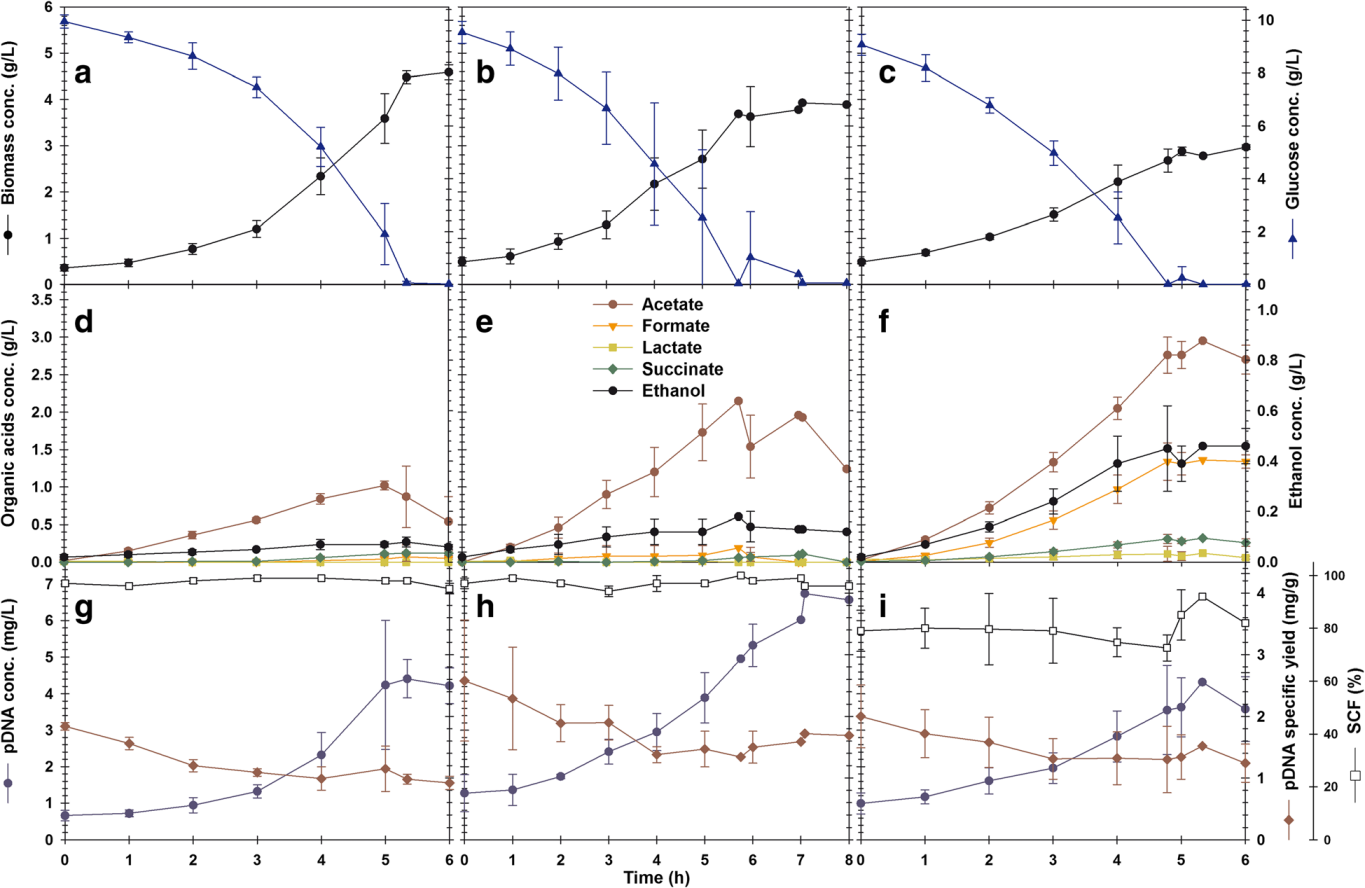
Growth, byproducts accumulation and pDNA production in the cultures. **a**-**c** Biomass and glucose concentration; **d**-**f** byproducts concentration; **g**-**i** pDNA concentration, yield and SCF. *Left panel*: aerobic cultures; *middle panel*: microaerobic cultures; *right panel*: oscillated DOT cultures. *Error bars* correspond to the standard deviation form 3 independent cultures

The growth, extracellular metabolite level, and pDNA concentrations for all cultures are depicted in Fig. 2. The kinetic and stoichiometric parameters are shown in Table 1. The biomass reached was lower for microaerobic and oscillated cultures than for aerobic conditions (Fig. 2a-c). Such less efficient biomass synthesis was also reflected in the growth rate and biomass yield on glucose (Y_X/S_), as seen in Table 1. Constant or cyclic oxygen limitation also increased byproduct accumulation (Fig. 2d-f). In aerobic cultures, acetate was the major byproduct, followed by traces of succinate and ethanol. In microaerobic cultivations, acetate was twice as abundant in aerobic cultures, and ethanol was produced significantly. Under oscillating DOT, 30% and 250% more acetate and ethanol were accumulated than under microaerobic cultures. DOT oscillations resulted in the high accumulation of formate, while succinate and lactate were produced scarcely (Fig. 2d-f, Table 1). The specific production rates of these byproducts were also increased as a result of oxygen limitations (Table 1). The specific rates of O_2_ uptake (${q}_{O_2}$) and CO_2_ generation (${q}_{CO_2}$) were highest in aerobic cultures. However, such rates were 30% and 87% lower in microaerobic and oscillated DOT cultures, respectively (Table 1).

**Table 1:** Exponential growth kinetic, stoichiometric parameters, carbon (CB) and degree of reduction (DRB) balances

Parameter	Units	Aerobic	Microaerobic	Oscillated
*μ*	h^−1^	0.49 ± 0.01	0.36 ± 0.03	0.36 ± 0.04
Y_X/S_	g/g	0.42 ± 0.01	0.34 ± 0.04	0.26 ± 0.03
Y_ACE/S_	g/g	0.10 ± 0.03	0.22 ± 0.04	0.32 ± 0.03
Y_FOR/S_	g/g	0.00 ± 0.00	0.04 ± 0.07	0.16 ± 0.01
Y_LAC/S_	g/g	0.00 ± 0.00	0.00 ± 0.00	0.01 ± 0.01
Y_SUC/S_	g/g	0.02 ± 0.00	0.00 ± 0.01	0.04 ± 0.01
Y_ETH/S_	g/g	0.01 ± 0.00	0.01 ± 0.01	0.05 ± 0.01
Y_O2/S_	mmol/g	12.2 ± 0.9	10.6 ± 1.2	1.2 ± 0.1
Y_CO2/S_	mmol/g	10.7 ± 0.7	8.1 ± 0.9	1.2 ± 0.1
*q*_*S*_	g/g h	1.15 ± 0.04	1.07 ± 0.15	1.39 ± 0.17
*q*_*ACE*_	g/g h	0.11 ± 0.03	0.23 ± 0.05	0.43 ± 0.04
*q*_*FOR*_	g/g h	0.00 ± 0.00	0.04 ± 0.07	0.22 ± 0.03
*q*_*LAC*_	g/g h	0.00 ± 0.00	0 ± 0	0.02 ± 0.01
*q*_*SUC*_	g/g h	0.02 ± 0.00	0 ± 0.01	0.05 ± 0.01
*q*_*ETH*_	g/g h	0.00 ± 0.01	0.01 ± 0.01	0.07 ± 0.02
OTR	mmol/L h	60.0 ± 2.5	40.6 ± 2.1	35.9 ± 3.7
CER	mmol/L h	52.4 ± 2.5	32.9 ± 3.1	26.9 ± 4.4
${q}_{O_2}$	mmol/g h	14.0 ± 0.6	11.2 ± 0.3	1.6 ± 0.1
${q}_{C{ O}_2}$	mmol/g h	12.2 ± 0.5	8.5 ± 0.8	1.7 ± 0.1
RQ	mmol/mmol	0.93 ± 0.03	0.76 ± 0.06	1.08 ± 0.04
CB	C-mol/C-mol	0.96 ± 0.01	0.92 ± 0.12	0.89 ± 0.04
DRB	e/C-mol	−0.12 ± 0.07	−0.06 ± 0.05	0.46 ± 0.16

The data represent the average ± standard deviation of 3 independent experiments

### pDNA production and quality under constant and oscillating DOT conditions

The pDNA yields on biomass (*Y*_*P/X*_) during the cultures are shown in Fig. 2g-i and are summarized in Table 2. Compared with aerobic cultures, *Y*_*P/X*_ was 30% higher under constant oxygen limitation and was similar to the oscillated case. Neither the pDNA-specific production rate (*q*_*P*_) nor pDNA global productivity (*Q*_*P*_) was significantly influenced by oxygen limitation (Table 2).

**Table 2:** pDNA production parameters

Parameter	Units	Aerobic	Microaerobic	Oscilated
P	mg/L	4.9 ± 0.7	5.5 ± 1.1	3.8 ± 1.0
*Y*_*P/X*_	mg/g	1.3 ± 0.0	1.8 ± 0.3	1.6 ± 0.4
*q*_*P*_	mg/g h	0.63 ± 0.00	0.64 ± 0.04	0.55 ± 0.12
Q_P_	mg/g h	0.17 ± 0.03	0.23 ± 0.02	0.25 ± 0.07
SCF	%	98 ± 0	97 ± 0	80 ± 6

*P* pDNA titer at the end of cultivation, *Y*_*P/X*_ average of the individual pDNA specific yield values during exponential growth, *q*_*P*_ pDNA specific production rate, *Q*_*P*_ pDNA global productivity, *SCF* average of the individual supercoiled pDNA fraction values during exponential growth. The data represent the average ± standard deviation of 3 independent experiments

**Fig. 3:**
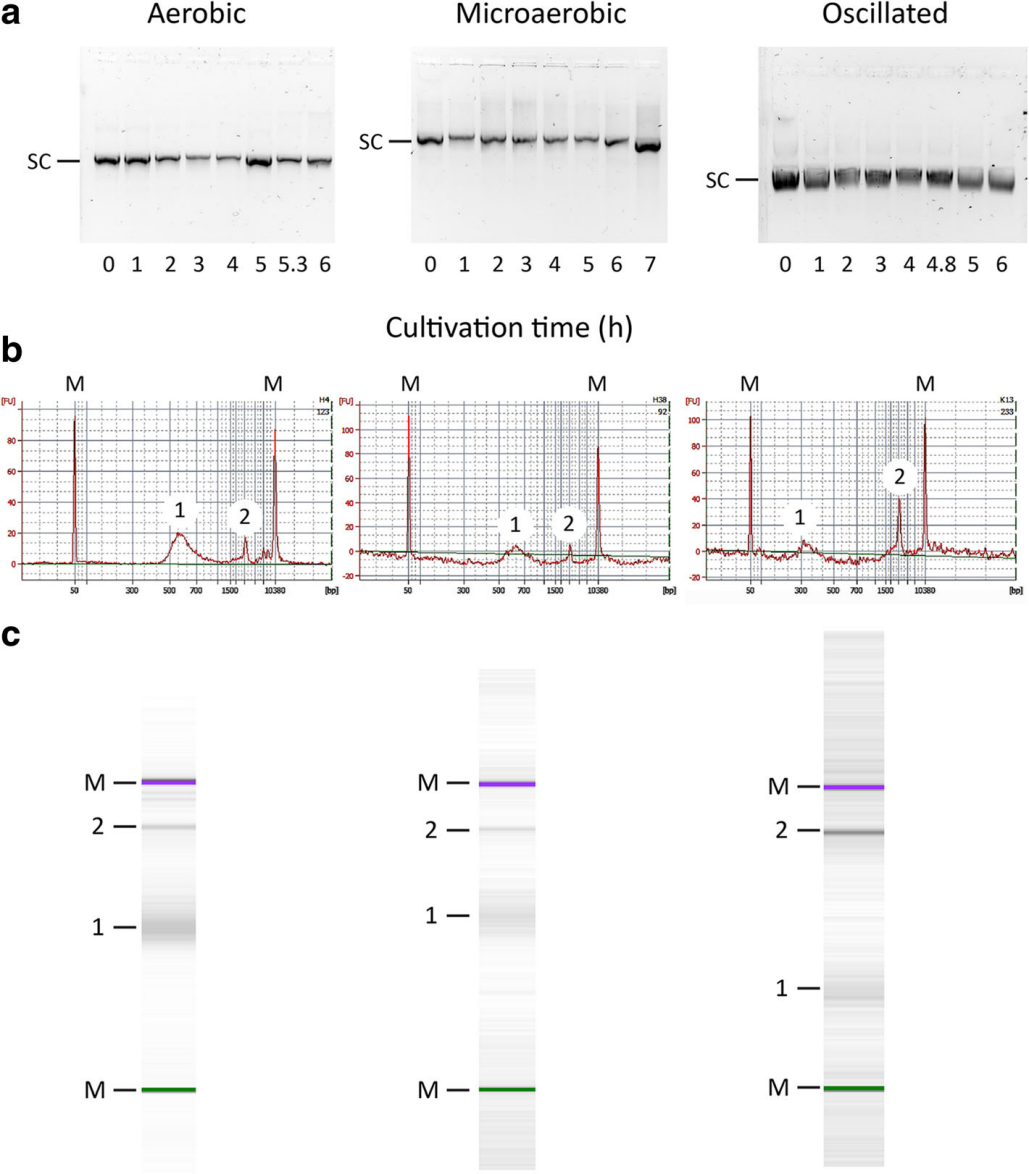
pDNA topological analyses. **a** Agarose gel electrophoresis images of pDNA samples through the cultivation time; **b** Chip electrophoresis electropherograms; **c** virtual gel agarose electrophoresis images of pDNA samples from the end of the cultures. *SC* indicates the pDNA supercoiled fraction; *M* denotes DNA markers; *1* and *2* indicate the two major pDNA populations found

Under aerobic and microaerobic conditions, the pDNA SCF remained close to 98% through the cultures (Fig. 2g and h). By contrast, when DOT was oscillated, SCF dropped to 80% (Fig. 2i) and showed more heterogeneity. This was further analyzed using chip electrophoresis (Fig. 3). Figure 3a shows the agarose gels, and Fig. 3b shows the electropherograms of chip electrophoresis. In the three electropherograms, two peaks are relevant. The first, at 3 kb, corresponds to the main supercoiled pDNA population, as indicated by Ding and coworkers [17]. The second peak appears at 0.6 kb in aerobic and microaerobic cultivations but at 0.3 kb in the oscillated cultures. The origin of these smaller DNA molecules in the oscillated culture is not clear, and they were not observed in the agarose electrophoresis (Fig. 3a). Moreover, the pDNA produced under oscillating DOT presented more and broader spikes in the electropherogram (Fig. 3b), and more bands in the virtual gel of chip electrophoresis (Fig. 3c) than the ones of aerobic and microaerobic cultures.

Plasmid sequencing and alignment analyses against the reported pVAX1 sequence revealed five nucleotide changes in plasmids produced in all conditions, as depicted in Additional file 1: Figure S1. A gap and a base replacement occurred around the kanamycin resistance cassette. Within and downstream the ColE1 replicon were found two additional base replacements. One last base replacement was located in the CMV promoter enhancer sequence. Such changes were found by the three alignment tools described in the Methods section.

### Estimation of the metabolic fluxes under constant and oscillating DOT conditions

The flux distribution calculations are based on the reactions present in the metabolic network and experimental uptake and production rates. In the FBA approach, the steady state has to be achieved. During exponential growth of batch cultures, it is generally assumed that intracellular fluxes are constant; thus, a quasi-stationary state is considered, and the FBA approach is valid. In the case of the calculation of the flux distribution for the aerobic and microaerobic conditions, FBA can be applied considering the exponential growth phase. Nevertheless, extending this methodology to the oscillated case complicates its application and validity because no constant growth rate is present. An attempt was made to simulate the oscillated growth condition of this strategy by assuming a constant growth rate. However, no feasible solution was obtained under the constraints for biomass or ${q}_{O_2}$ maximization, and the prediction error range was between 20 and 70%. Better predictions of *μ* were obtained, with errors of less than 11%, by incorporating appropriate restrictions according to the scenarios and tuning the gas flux boundary constraints (Additional file 2: Table S1). Response surfaces were obtained for the sensitivity analysis for the aerobic, microaerobic and oscillated scenarios (Additional file 3: Figure S2). The topology of such surfaces was quite similar for the aerobic and microaerobic cases. By contrast, the topology of the oscillated case surface was remarkably different from the others. Notwithstanding, the response surfaces indicate that good estimations can be expected within the range of the experimental extracellular rates of the present work.

**Fig. 4:**
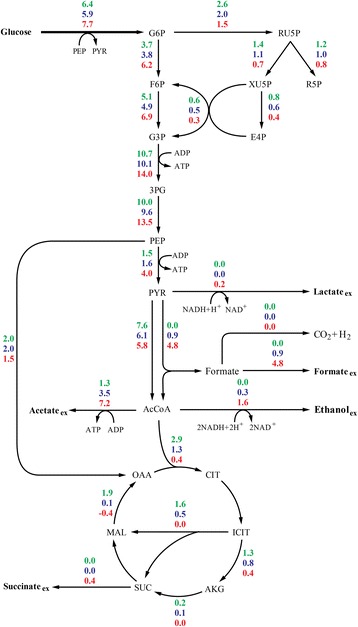
Estimated metabolic fluxes distribution. Values correspond, from top to bottom, to aerobic, microaerobic and oscillated cultures

The estimated fluxes resulting from these strategies are depicted in Fig. 4. The estimated flux to the pentose phosphate pathway was found to be 20% and 40% lower under microaerobic and oscillating DOT conditions, respectively in comparison to aerobic cultures. The glycolytic fluxes were also similar for aerobic and microaerobic conditions but were increased by 31% when DOT was oscillated (Fig. 4). The flux through the pyruvate dehydrogenase decreased with low or intermittent oxygen availability, a finding that is coincident with the flux of pyruvate to byproducts. Similar results were obtained in the case of citrate synthase. In fact, the TCA cycle is estimated to work in a closed mode under aerobic and microaerobic (albeit less active) conditions [18]. Nevertheless, the estimated fluxes point to a predominantly branched TCA cycle when DOT was oscillated (Fig. 4).

**Fig. 5:**
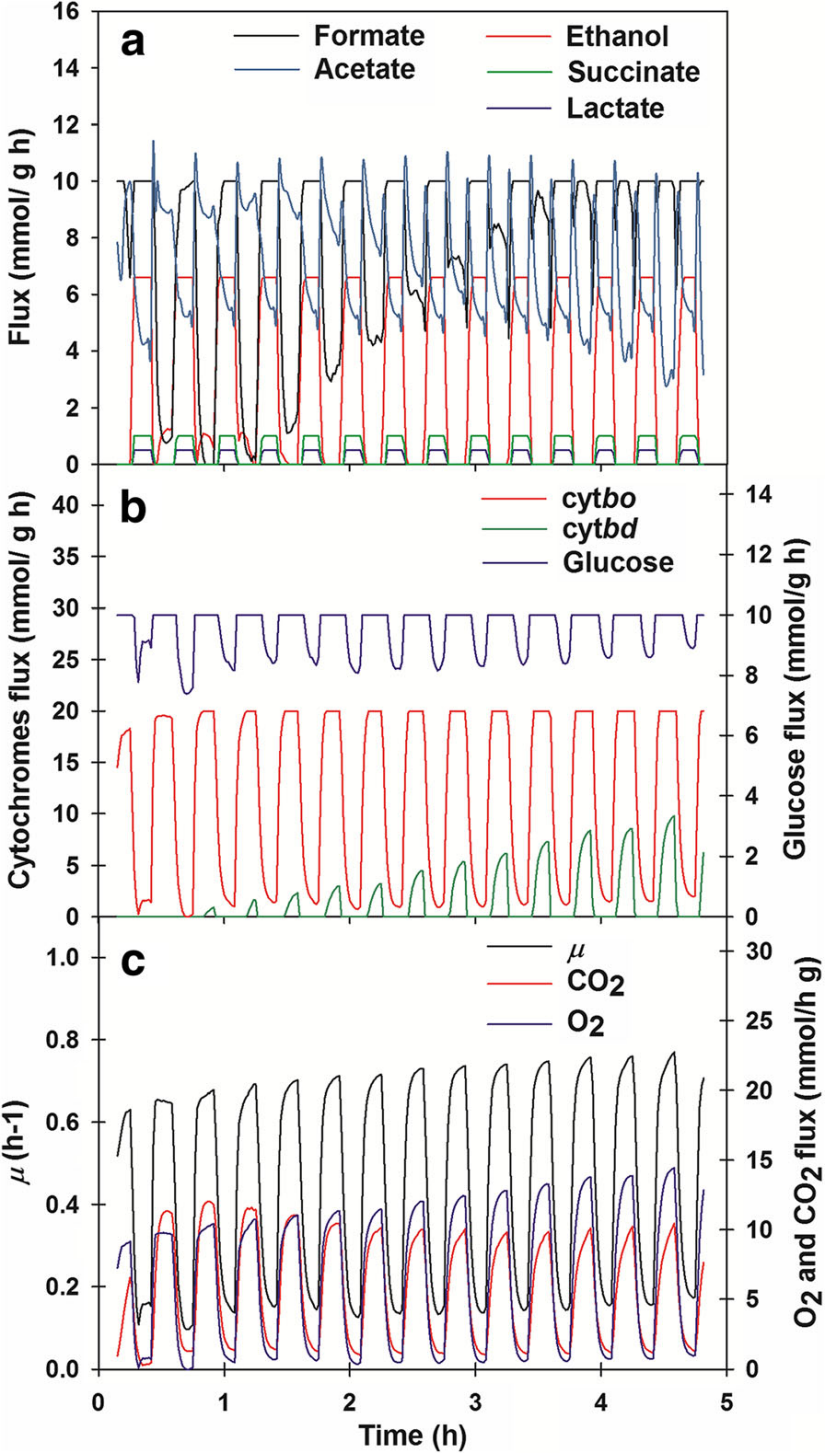
Estimated flux through the exponential growth phase of cultures under oscillated DOT. **a** Mixed-acid fermentation products fluxes; **b** glucose fluxes and fluxes through the cytochromes; **c** O_2_ and CO_2_ fluxes and *μ* evolution

To overcome the unsteady state of the oscillatory growth condition, an extended approach of FBA was considered for the dynamics (dFBA). In this approach, the bounds needed for the metabolic model were calculated solving a system of ordinary differential equations that accounted for the concentration profile of the extracellular metabolites [19, 20]. The calculations related the metabolic network state to the changes in the environmental (bioreactor) variables at each time. Based on this approach, for the oscillatory case in this study, we calculated the O_2_ and CO_2_ rates at each time-point, and these values were used as bound for the metabolic network model. The estimated instantaneous (time-point)${q}_{O_2}$and ${q}_{CO_2}$ from an oscillated cultivation were used as inputs to simulate biomass maximization based on the assumptions in dFBA. The results are shown in Fig. 5. As a result, it can be seen that glucose and cytochrome fluxes (Fig. 5b), as well as *μ* (Fig. 5c), oscillated in phase with gas fluxes. By contrast, fermentation fluxes (Fig. 5a) were out of phase; their values were high when gas fluxes values were low. Formate flux had a different response; during the first hour, low fluxes almost fell to zero, but later began increasing up to 4 h, diminishing drastically its amplitude. Acetate also had a distinctive oscillation pattern; flux increased sharply at the onset of transitions but then slowed down within the stationary periods, although never reached zero. Flux through cytochrome *bo* oscillated at a nearly constant amplitude. By contrast, flux through cytochrome *bd* displayed increasing amplitude (Fig. 5b).

### Potential targets for genetic modification assessed through in silico experiments

In order to find possible targets for strain improvement, metabolic fluxes were simulated for biomass maximization under the constraints for microaerobic culture. The mutations depicted in Table 3 were predicted to strongly impact the flux through the ribose-5-phosphate isomerase. Such enzyme converts ribulose-5-phosphate to ribose-5-phosphate, which is a building block for nucleic acids synthesis. For the purpose of these in silico experiments, the ribose-5-phosphate yield (Y_r5p_) is defined as the ratio of the flux thorough the ribose-5-phosphate isomerase to the flux to the biomass objective functions. Eliminating the flux through the pyruvate dehydrogenase (PDH) resulted in a 4.84-fold increase of Y_r5p_ and no change on the specific growth rate compared with the original background. Higher increases of Y_r5p_ are predicted if LDH (lactate dehydrogenase), isocitrate lyase (ICL) genes or malate synthase (MALS) are combined with the PDH mutation (Table 3). However, such double mutants are predicted to grow at a lower rate than the wild-type strain (Table 3). In contrast, eliminating the fluxes through the glutaminase (GLUN, which converts L-glutamine to L-glutamate and water), results in an increase of both the specific growth rate and Y_r5p_, compared to the wild type strain (Table 3).

**Table 3:** Predicted effect of single and double mutations on the specific growth rate (*μ*) and ribose-5-phosphate yield (Y_r5p_) under microaerobic conditions

Mutations	*μ* (h^−1^)	Y_r5p_ (−)
PDH	0.37	4.83
PDH, LDH	0.22	6.15
PDH, ICL	0.22	6.15
PDH, MALS	0.22	6.15
PDH, GLUN	0.42	3.54

Simulations were performed to maximized biomass while fixing to zero the flux through the following enzymes: *ICL* Isocitrate lyase, *LDH* Lactate dehydrogenase, *MALS* Malate synthase, *GLUN* Glutaminase

## Discussion

Bioreactor scale-up often results in lower global mixing efficiency than bench-scale bioreactors. As a consequence, substrates and dissolved gases are heterogeneously distributed. The physiological adaptation of cells to heterogeneous conditions has been intensively studied yet it is far from being well understood. More studies focusing on the quality of a given product are needed to assess the process robustness. One of the key parameters affecting the physiology of *E. coli* is the availability of oxygen because it causes important metabolic rearrangements. While several previous studies have focused on the impact of oxygen availability on *E. coli*, only a few have studied the relevance of this parameter on product quality. For example, it has been demonstrated that oxygen limitation contributes to the synthesis of the non-canonical amino acid norvaline by *E. coli* [21, 22]. This may affect the quality of the recombinant protein produced. In the present study, DOT heterogeneities were simulated by transitions in the stirring rate, (similar to previous studies by Brognaux et al. [23], and Han et al., [24]) and were compared with cultures under constant oxygen availability. Specifically, we measured the plasmid production capabilities of *E. coli* in three scenarios in relation to the oxygen availability, aerobic, microaerobic and oscillatory DOT conditions. The observed accumulation of mixed acid fermentation products and variations in *μ* and *Y*_*x/s*_ with respect to aerobic cultures are well known consequences of microaerobic conditions. Under microaerobic conditions, the observed decrease of ${q}_{O_2}$and ${q}_{CO_2}$ is evidence of the oxygen limitation at DOT = 3% air sat. By contrast, the oscillatory transit of the cell between aerobicity and oxygen limitation lead to a sharp drop in ${q}_{O_2}$and ${q}_{CO_2}$ but an RQ greater than 1, indicating the prevalence of a fermentative rather than a respiratory catabolism of the glucose. This brought duplication of acetate production compared with the microaerobic case, a strategy used by the cell to maximize ATP production. This may occur due to very fast accumulation of organic acids when oxygen is totally depleted, as reported previously [5]. On the other hand, high-speed agitation periods increased DOT by enhancing oxygen transfer and quickly restored respiratory metabolism. Nonetheless, the fermentative enzymes remained inactive in the presence of oxygen. Carbon and redox balances for cultures under oscillatory DOT did not close as satisfactorily as those for aerobic and microaerobic cultures (Table 1). It is likely that some metabolites not measured, such as pyruvate or CO_2_ in the liquid phase or ethanol in the gas phase, affected the overall balance.

The estimated metabolic fluxes under microaerobic conditions were consistent with those previously reported using *E. coli* W3110 and similar growth conditions [25]. The estimated lower flux to the pentose phosphate pathway and TCA cycle agrees with the observed reduction in the growth rate and biomass synthesis compared with that in aerobic cultures. Despite such lower fluxes (closely related to DNA building block synthesis and energy generation), the values of *Y*_*p/x*_ were greater under microaerobic than under aerobic conditions. This effect on *Y*_*p/x*_ coincides with previous reports, which attributed such an increase to a reduction in the growth rate under microaerobic conditions [9]. Therefore, the ATP generation from acetate synthesis could play an important role in the synthesis of pDNA under oxygen limitation. Glyoxylate bypass was estimated to be active under aerobic conditions, coincident with previous studies using the strain W3110 [26]. Furthermore, fluxes through the glyolxylate bypass decreased upon oxygen limitation in accordance to experimental evidence [27], rendering glyoxylate bypass completely inoperative under oscillated DOT (Fig. 4).

To overcome the lack of a (*quasi*) steady-state under oscillating DOT, we used exhaust gas monitoring to estimate O_2_ and CO_2_ fluxes to obtain feasible solutions for maximizing biomass at each time. The results of this time-point approach yielded reasonable changes in the fermentation metabolite production rates. As expected, byproducts were low at periods of high O_2_ flux and vice versa. Acetate flux was less accurately predicted, and formate formation was inconsistent because almost all times were different from zero. Fluxes through the cytochromes *bo* and *bd* were also evaluated. Cytochrome *bo* responded fairly well to O_2_ flux changes, but not cytochrome *bd*, which behaved more like an extra supply rather than a backup for respiratory activity that was expected to switch on once cytochrome *bo* flux was low [28, 29]. We did not incorporate a regulatory approach into our model to account for oxygen limitation inhibition for cytochrome *bo* and pyruvate formate lyase fluxes (as in [20]). Therefore, the discrepancies found only reflect the limitations of the model and strategy used to evaluate it, but they were still acceptable due to the good results obtained by classical FBA and a reduced metabolic network. Undoubtedly, refinement of the tools and of the model are needed to overcome such limitations. Moreover, the obtained results can guide the experimental effort and help to identify targets for cell engineering. Eliminating some of the fermentative pathways could be viable if respiratory activity could be sustained for prolonged O_2_ limitation [25].

The production of pDNA was nearly unaffected by a constant low DOT. This opens the possibility for the microaerobic production of pDNA, which may be viable if strains with a lower accumulation of by-products and increased respiratory capacity are available [25]. By contrast, under oscillatory oxygen limitation, pDNA production was lower. However, such a decrease was not at the same level because it was reported using anaerobic shifts [7]. Nonetheless, these differences may be the result of using different *E. coli* K-12 derivative strains and plasmids. Hopkins and coworkers [7] used the *E. coli* AB1157 strain to replicate an RK6 replicon plasmid. Namdev and coworkers [8] used the *E. coli* DH5α strain, a highly mutated background commonly used for pDNA amplification, to replicate a pUC replicon plasmid. In our work, we employed a much less mutated background to also amplify a pUC replicon plasmid. This opens the question of whether such discrepancies have any relation to the plasmid-host interactions.

The pDNA topology was strongly affected by DOT oscillations. In the model strain, the degree of pDNA supercoiling is mainly determined by the activities of topoisomerase I (an IA type topoisomerase) and gyrase (a IIA topoisomerase) [30, 31]. Because gyrase introduces negative supercoils at the expense of ATP hydrolysis, its activity is connected to the [ATP]/[ADP] ratio. Furthermore, it has been demonstrated that the ratio correlates with the degree of pDNA supercoiling [32, 33]. Hsieh and coworkers [32] found that, upon an anaerobic shift, the adenylate pool rapidly drops and concomitantly relaxes DNA in less than 15 min, rendering pDNA heterogeneity. In transitions from anaerobiosis to aerobiosis, the plasmid becomes heterogeneous and relaxed within 5 min. Nonetheless, it becomes homogeneous but with a lower degree of negative supercoiling than before the shift after 15 min. Although the [ATP]/[ADP] ratio is expected to be lower under the microaerobic than under the aerobic conditions studied in the present report, SCF was similar between them. The energy metabolism is more affected under oscillating DOT. Accordingly, a strong decrease in the SCF was observed for these cultures, as well as higher heterogeneity evidenced by chip electrophoresis. A detailed analysis of the supercoiling density may be very useful to better understand this fact and to design pDNA backbones more robustly for industrial applications [34].

The impact of the process conditions on pDNA sequence, which can be a major issue, is still poorly documented. For instance, a recent study reported the contamination by 1.3 kb IS2 in a pDNA vaccine candidate, with pVAX1 as the backbone, between the kanamycin resistance cassette and BGH polyA signal [35]. The frequency at which such undesirable events could be triggered relies on the presence of switch sites of GC and AT skews (ssGCs and ssATs) in pDNA, as well as on the time, cell density and mode of cultivation [36]. Complete sequencing of the plasmids from the master cell bank and from the conditions assessed in this work demonstrated that DOT availability did not altered plasmid sequence fidelity. The nucleotide changes detected in plasmids from all conditions were the same and might were already present in the pVAX1 plasmid extracted from the master cell bank. The changes occurred with major incidence around the origin of replication and kanamycin resistance cassette. These results should be taken cautiously because the number of base changes detected fall within the error rate of the technique [37]. Dedicated experiments to better clarify this issue and its underlying mechanisms should be performed.

Estimation of the metabolic fluxes could be useful for the design of strains that better cope with environmental limitations. The network used in the present work was used to estimate fluxes that could lead to maximum biomass synthesis. Eliminating the flux through the pyruvate dehydrogenase complex was found to increase the flux through the pentose phosphate pathway (PPP) and thus the synthesis of ribose-5-phosphate, as evidenced by the Y_r5p_ (Table 3). This could allocate more building blocks for DNA synthesis, while the AcCoA would be synthesized mainly from PEP. Increasing the flux to the PPP efficiently reduced the metabolic burden in aerobic cultures of *E. coli* bearing a high copy number plasmid [38]. Together with the mutations of the glyoxylate shunt (ICL and MALS) elimitaing the flux through the PDH further increased the predicted Y_r5p_. The underlying rationale of these results might be to eliminate as much as possible the redundant enzymatic activities that scavenge ATP, NADH or NADPH. Since the network used for these simulations did not include specific reaction for pDNA synthesis, it is not possible to estimate whether pDNA yields would increase in the proposed mutants.

## Conclusions

Microaerobic conditions shifted the metabolism of *E. coli* to a predominantly fermentative mode. This was more evident in the case of oscillatory DOT. However, microaerobic conditions had little effect on pDNA production and quality. Therefore, the microaerobic production of pDNA may be a viable alternative, provided that engineered cells are available with a better metabolic performance. The estimated TCA and pentose phosphate pathway fluxes can help to identify cell engineering targets for such a task. Our results suggest that DOT heterogeneities may increase the topological heterogeneity pDNA and that oxygen availability does not influence pDNA sequence. This should be considered during bioreactor operation at several scales and for better strain and vector design.

## Nomenclature

- *μ* - Specific growth rate (h^−1^)
- Y_X/S_ - Biomass on glucose yield (g/g)
- Y_ACE/S_ - Acetate on glucose yield (g/g)
- Y_FOR/S_ - Formate on glucose yield (g/g)
- Y_LAC/S_ - Lactate on glucose yield (g/g)
- Y_SUC/S_ - Succinate on glucose yield (g/g)
- Y_ETH/S_ - Ethanol on glucose yield (g/g)
- Y_O2/S_ - Oxygen on glucose yield (mmol/g)
- Y_CO2/S_ - Carbon dioxide on glucose yield (mmol/g)
- *q*_*S*_ - Glucose specific uptake rate (g/g h)
- *q*_*ACE*_ - Acetate specific production rate (g/g h)
- *q*_*FOR*_ - Formate specific production rate (g/g h)
- *q*_*LAC*_ - Lactate specific production rate (g/g h)
- *q*_*SUC*_ - Succinate specific production rate (g/g h)
- *q*_*ETH*_ - Ethanol specific production rate (g/g h)
- OTR - Volumetric oxygen transfer rate (mmol/L h)
- CTR - Volumetric carbon dioxide transfer rate (mmol/L h)
- ${q}_{O_2}$ - Oxygen specific uptake rate. (mmol/g h)
- ${q}_{C{ O}_2}$ - Carbon dioxide specific production rate (mmol/g h)
- RQ - Respiratory quotient (mmol/mmol)
- CB - Carbon balance (C-mol/C-mol)
- DRB - Degree of reduction balance (e/C-mol)
- P - Plasmid DNA titer (mg/L)
- *Y*_*P/X*_ - pDNA specific yield (mg/g)
- *q*_*P*_ - pDNA productivity (mg/g h)
- Q_P_ - pDNA global productivity (mg/g h)
- SCF - pDNA supercoiled fraction (%)
- *p* - Absolute pressure of the gaseous stream (bar)
- *F* - Volumetric flow rate of the gaseous stream (L/h)
- R - Ideal gas constant (0.0821 bar L K^−1^ mol^−1^)
- *V*_*L*_ - Liquid volume (L)
- *T* - Temperature of the gaseous stream (K)
- ${y}_{O_2}$ - Oxygen molar fraction in the gaseous stream (mmol/mmol)
- ${y}_{CO_2}$ - Carbon dioxide in the gaseous stream (mmol/mmol)
- ${y}_{H_2 O}$ - Water molar fraction in the gaseous stream (mmol/mmol)
- *R*_*I*_ - Inert ratio (mmol/mmol)
- *i* - Inlet
- *o* - Outlet
- *X* - Biomass concentration (gCDW/L)
- DOT - Dissolved oxygen tension (%)
- FBA - Flux balance analysis
- dFBA - Dynamic flux balance analysis
- CMV - Cytomegalovirus

## Additional files

Additional file 1: Prediction of *μ* under the different oxygen availabilities. (TIFF 512 kb)

Additional file 2: Base changes found in pVAX1. (DOCX 16 kb)

Additional file 3: Response surfaces for the sensitivity analysis. (TIFF 12468 kb)
