# Supplementary figures and images for: Heterogeneous oxygen availability affects the titer and topology but not the fidelity of plasmid DNA produced by Escherichia coli
Source: BMC Biotechnol. 2017 Jul 4;17:60. doi: 10.1186/s12896-017-0378-x (PMC5496438; doi:10.1186/s12896-017-0378-x)

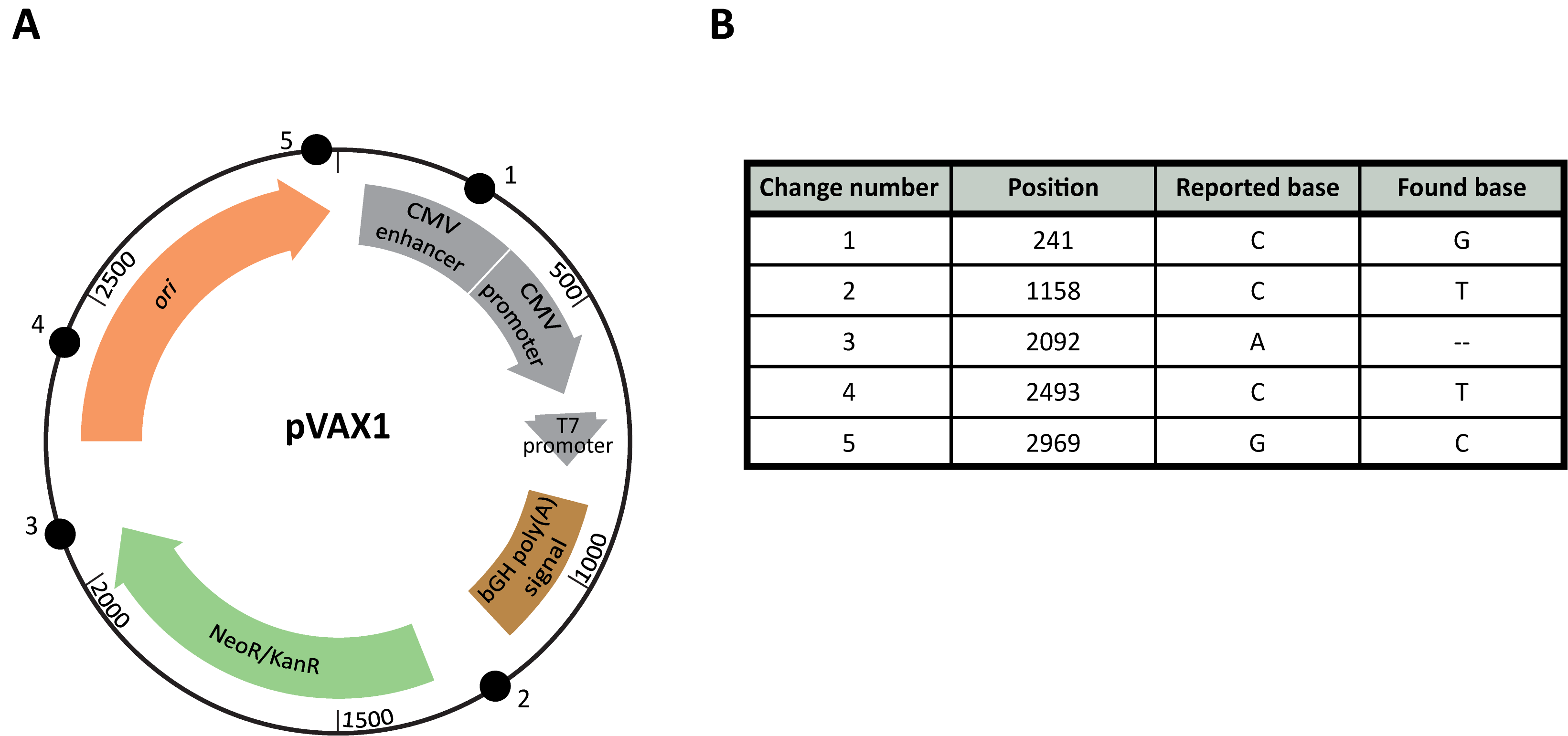

Supplement: Supplementary file 1 — Prediction of μ under the different oxygen availabilities. (TIFF 512 kb) [file 12896_2017_378_MOESM1_ESM.tif]

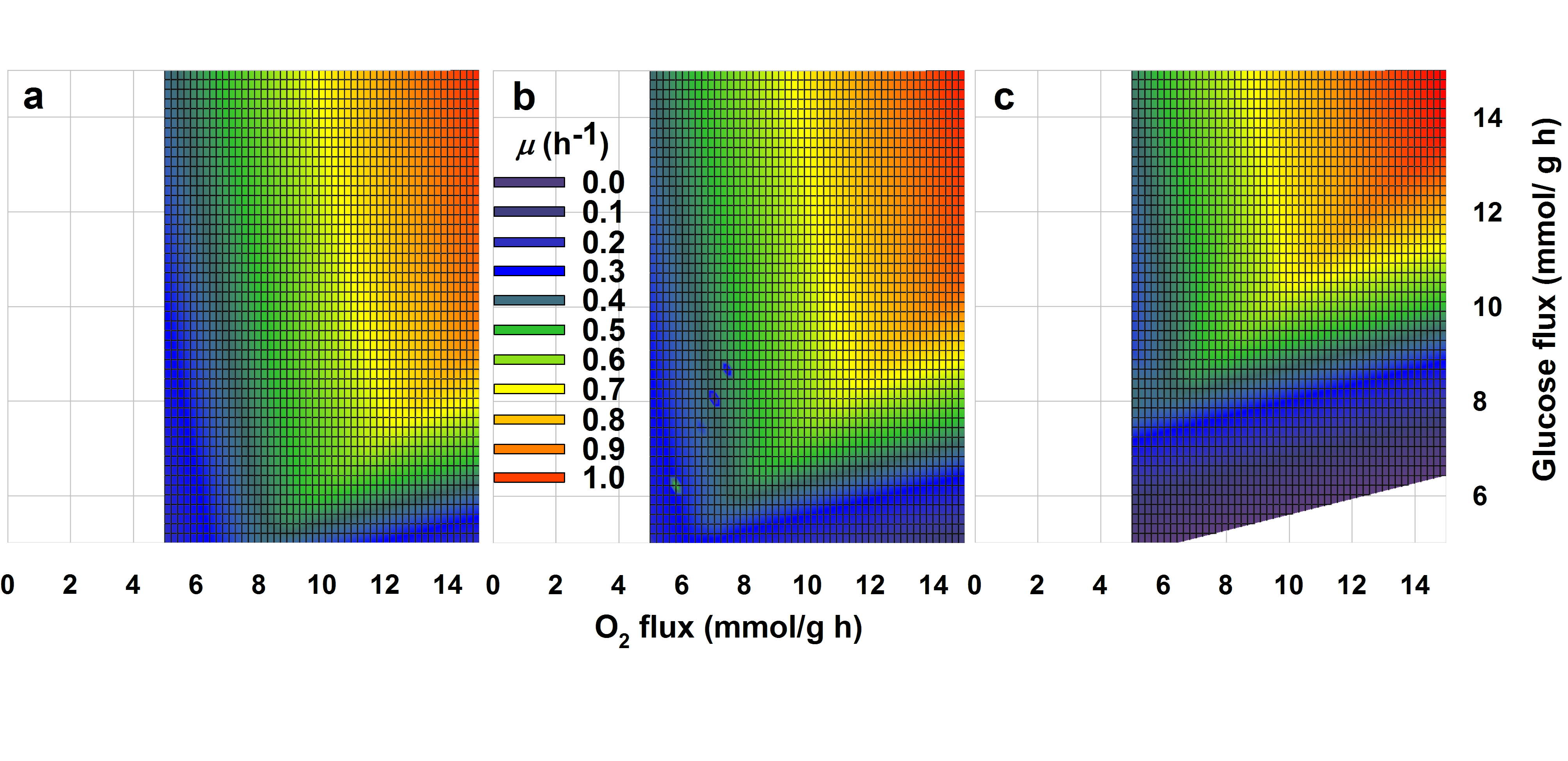

Supplement: Supplementary file 3 — Response surfaces for the sensitivity analysis. (TIFF 12468 kb) [file 12896_2017_378_MOESM3_ESM.tif]
