# Supplementary material for: Heterogeneous oxygen availability affects the titer and topology but not the fidelity of plasmid DNA produced by Escherichia coli
Source: BMC Biotechnol. 2017 Jul 4;17:60. doi: 10.1186/s12896-017-0378-x (PMC5496438; doi:10.1186/s12896-017-0378-x)
Supplement: Supplementary file 2 — Base changes found in pVAX1. (DOCX 16 kb) [file 12896_2017_378_MOESM2_ESM.docx]

| Simulation | Parameter | Aerobic | | Microaerobic | | Oscillated | |
| --- | --- | --- | --- | --- | --- | --- | --- |
|  |  | Exp | In-silico | Exp | In-silico | Exp | In-silico |
| Max  Biomass | Zbiomass/  *μ* | 0.49 | 0.28 | 0.36 | 0.11 | 0.36 | NFS |
| Max  O_2_ | EX_o2(e)/  *qO_2_* | 14 | 11.8 | 11.17 | 8.52 | 1.61 | NFS |
|  | EX_co2(e)/  *qCO_2_* | 12.2 | 12.2 | 8.53 | 8.53 | 1.73 | NFS |
|  | RQ | 0.87 | 1.00 | 0.76 | 1.00 | 1.07 | NFS |
| Max Biomass + opening O_2_ and CO_2_ | Zbiomass/  *μ* | 0.49 | 0.56 | 0.36 | 0.43 | 0.36 | 0.39 |
|  | EX_o2(e)/  *qO_2_* | 14 | 10.4 | 11.17 | 7.65 | 1.61 | 5.73 |
|  | EX_co2(e)/  qCO_2_ | 12.2 | 11.1 | 8.53 | 7.99 | 1.73 | 5.17 |
|  | RQ | 0.87 | 1.07 | 0.76 | 1.04 | 1.07 | 0.90 |
| Improved  Max Biomass + opening O_2_ and CO_2_ | Zbiomass/  *μ* | 0.49 | 0.47 | 0.36 | 0.39 | 0.36 | 0.37 |
|  | EX_o2(e)/  *qO_2_* | 14 | 12 | 11.17 | 8.53 | 1.61 | 6.75 |
|  | EX_co2(e)/  *qCO_2_* | 12.2 | 12 | 8.53 | 8.53 | 1.73 | 6.17 |
|  | RQ | 0.87 | 1.00 | 0.76 | 1.00 | 1.07 | 0.91 |

For the improved maximization of biomass + opening O_2_ and CO_2_ simulation, in the aerobic scenario, the limits of $q_{O_{2}}$ and $q_{{CO}_{2}}$ were tightened between 14-12 mmol /g h, and 12 mmol/ g h, respectively. In the microaerobic scenario, the limits of $q_{O_{2}}$ and $q_{{CO}_{2}}$ tightened between 8.53-14 mmol/g h, and 8.53 mmol/g h, respectively. For the oscillated scenario, the reaction of the cytochrome *bo* was constrained to 1 mmol/g h, and the limits of $q_{O_{2}}$ and $q_{{CO}_{2}}$fixed to 20 mmol/g h, respectively. NFS, No Feasible Solution.
